# Ca^2+^ signaling in HCO_3_^−^ secretion and protection of upper GI tract

**DOI:** 10.18632/oncotarget.21840

**Published:** 2017-10-12

**Authors:** Jialin He, Xin Yang, Yanjun Guo, Fenglian Zhang, Hanxing Wan, Xuemei Sun, Biguang Tuo, Hui Dong

**Affiliations:** ^1^ Department of Gastroenterology, Affiliated Hospital, Zunyi Medical College, Zunyi, China; ^2^ Department of Gastroenterology, Xinqiao Hospital, Third Military Medical University, Chongqing, China

**Keywords:** calcium signaling, intestinal epithelial, HCO_3_^−^ secretion, upper gastrointestinal protection

## Abstract

The cytosolic calcium ([Ca^2+^]cyt) is one of the most important cell signaling that can modulate gastrointestinal (GI) epithelial secretion and promote GI mucosal wound repair. The GI mucosal bicarbonate secretion is the main mechanism of mucosal protection. Our research team has been working in this field and provided solid evidence for the important role of Ca^2+^ signaling in the regulation of GI epithelial secretion and the underlying molecular mechanisms. In this review, we attempt to systemically review the current status of our knowledge on the role of Ca^2+^ signaling in the regulation of intestinal bicarbonate secretion and in the upper GI epithelial protection. We expect that novel targets could be identified for drug development to better protect GI mucosa and treat mucosal injury with the advance in this filed.

## INTRODUCTION

Cytosolic free Ca^2+^ ([Ca^2+^]_cyt_) plays an essential role in a variety of mammalian cells through the regulation of many biological functions, including neurotransmitter release, muscle contraction, gene regulation, cell proliferation, and apoptosis [1]. Therefore, dysregulation of [Ca^2+^]_cyt_ homeostasis may result in pathological changes in many systems. Under physiological conditions, the function of Ca^2+^ as a cellular messenger is based on the presence of a concentration gradient between intracellular Ca^2+^ ([Ca^2+^]_i_) and extracellular Ca^2+^ [2], although different cell types combine different types of Ca^2+^ signaling to accomplish their specific physiological functions. Differences in Ca^2+^ signatures, which are key factors that determine specific Ca^2+^-dependent cellular responses, depend on complex, spatiotemporal variations in [Ca^2+^]_cyt_. A major determinant of these variations is based on functionally distinct calcium channels and exchangers. Ca^2+^ release from intracellular stores is mediated by ryanodine receptor (RyR) and inositol triphosphate receptor (IP3R) channels. RyRs are activated by a rise in [Ca^2+^]_i_, i.e., Ca^2+^-induced Ca^2+^ release (CICR) [3].

[Ca^2+^]_cyt_ also plays critical roles in the regulation of many biological functions in the digestive system [4, 5], such as nutrient digestion and absorption, epithelial ion transport and secretion, and gastrointestinal (GI) motility [6]. [Ca^2+^]_i_ is also an important factor that can accelerate GI epithelial wound repair. Bicarbonate (HCO_3_^−^) secretion in the intestinal mucosa is the main protective mechanism for the intestinal mucosal barrier. The ability of the duodenal mucosa to secrete mucus and bicarbonate combined with epithelial cell proliferation and migration constitutes an important protective mechanism for the intestinal mucosa.

The cAMP and cGMP signaling pathways play important roles in the regulation of intestinal bicarbonate secretion, and the details of the underlying mechanisms are well understood. Several good reviews have discussed this topic. However, little is known about the role of Ca^2^+ signaling in this regulation. Our research team has been working in this field to provide solid evidence for an important regulatory role of Ca^2+^ signaling and the underlying mechanisms. Therefore, in this review, we attempt to systemically review the current status of knowledge of the roles of calcium signaling in the regulation of intestinal bicarbonate secretion and upper GI protection.

### Intestinal HCO_3_^−^ secretion in GI protection

### Role of HCO_3_^−^ in the intestinal epithelium

The proximal portion of the duodenal lumen often attains acidity approaching a pH of 2 [7–9], but the pH remains neutral in the vicinity of the epithelial cell surface [10]. The pH gradient affects the formation of bicarbonate and mucus by epithelial cells. The viscoelastic mucosal gel at the epithelial surface and bicarbonate secretion to the mucosal gel provide a pre-epithelial damage defense mechanism [11]. The mucosal gel consists of 0–5% mucins (glycoproteins) and > 90% water [12]. Glycoproteins are secreted through exocytosis at the epithelial cell surface and the Bruner glands. Water provides continuous coverage of the gel on the epithelial cell surface.

Epithelial cells in the gastrointestinal tract remain in close proximity to one another by closing the top of the spaces between cells. In contrast to gastric epithelial cells, duodenal epithelial cells are commonly referred to as a “leaky” epithelium due to the high penetration of ions between the cells via electrolyte passive transport. The GI epithelium covers the largest surface area in the body. Because they are exposed to the external environment, skin cells are subjected to potentially destructive factors of exogenous and endogenous origin. Duodenal mucosal bicarbonate secretion is recognized as the main defense mechanism for the intermittent expulsion of gastric hydrochloric acid. HCO_3_^−^ is secreted in higher amounts by the duodenum than by areas such as the stomach and small intestine.

### Duodenal mucosal HCO_3_^−^ secretion

HCO_3_^–^ is secreted by the duodenal mucosa in the presence of hydrochloric acid and pepsin and intermittently exits the gastric duodenum to protect people from the pulsed key role. One unique function of the duodenal epithelium in the small intestine is the secretion of bicarbonate with a higher velocity, which enables it to reach the more distant parts of the mucosa. HCO_3_^−^ is secreted in response to major physiological stimuli, such as the presence of duodenal acid. Acid-induced HCO_3_^−^responsive medullary nerve pathways are involved in the release of vasoactive intestinal peptide (VIP), acetylcholine (ACh), and E-type prostaglandins (PGs) from mucosal epithelial cells [13]. VIP is a very potent peptide that stimulates intestinal HCO_3_^−^ secretion, and infusion of the VIP peptide increases duodenal mucosal transport of HCO_3_^−^ in all species [14–16]. Other mediators also stimulate duodenal bicarbonate transport, including cholecystokinin (CCK), pancreatic polypeptide, neurotensin, glucagon, pituitary adenylate cyclase activating peptide (PACAP), and angiotensin II [17–20]. Several reporter systems, including (iv) nitric oxide (NO) synthase (NOS), inhibit the addition of N-nitro-L-arginine methyl ester (L-NAME) to duodenal mucosal bicarbonate secretion [21–24].

Three major messenger system shave been implicated in the intracellular control of HCO_3_^−^ transport: i) intracellular calcium-induced responses (muscarinic M3 receptor agonists and CCKA), ii) cyclic adenosine monophosphate-activated transport (prostaglandin EP3 receptor agonists with dopamine D1 receptor agonists) and iii) cyclic GMP activation transport (uroguanylin, guanylin, and heat-stable enterotoxin).

The duodenum has different acid-base transport mechanisms that possibly reflect the activation of a second messenger system. HCO_3_^–^ and CO_2_ reach the epithelium via the blood, and HCO_3_^−^ is imported to the basolateral membrane via Na^+^(n)-HCO_3_^−^ cotransporters (NBCs). CO_2_ diffuses into enterocytes, and HCO_3_^–^ is formed intracellularly by carbonic anhydrase during the conversion of CO_2_ + H_2_O to HCO_3_^–^ + H^+^. Enterocytes export HCO_3_^−^ at the apical membrane via a Cl^–^/HCO_3_^–^ exchanger and an anion conductive pathway. The cystic fibrosis transmembrane conductance regulator (CFTR) has been suggested to function as a ubiquitous transmembrane channel for the transport of Cl ^-^ and HCO_3_^–^ [25–27].

### Ca^2+^ regulation of intestinal HCO_3_^–^ secretion

### Ca^2+^ signaling in intestinal epithelial cells

Most of the HCO_3_^–^ secreted by epithelial cells is in response to multiple inputs from hormones, neurotransmitters, and autacoids that evoke cytoplasmic Ca^2+^ signaling.

The increase in the cytoplasmic IP3 concentration stimulates the IP3 receptor (IP3R) in the endoplasmic reticulum (ER) and the rapid release of free Ca^2 +^ from the ER Ca^2 +^ store to the cytoplasm. Among the three IP3R paralogues (IP3R1-IP3R3), IP3R2 and IP3R3 are the major isomers in epithelial cells [28].

ER Ca^2+^ release is frequently followed by the activation of store-operated channels (SOCs) in the plasma membrane, such as the Orai [29–31] and transient receptor potential cation (TRPC) channels [32–34]. In response to the depletion of Ca^2+^ stores, the ER Ca^2+^ sensor stromal interaction molecule 1 (STIM1) clusters with and activates these SOCs [35, 36]. In some epithelial sites, such as the duodenal mucosa, Ca^2+^ entry via the reverse mode of the Na^+^/Ca^2+^ exchanger (NCX) can play a role in the sustained increase in [Ca^2+^]_i_ [37]. Finally, the increase in [Ca^2+^]_i_ activates the sarco/endoplasmic Ca^2+^ ATPase (SERCA) and plasma membrane Ca^2+^ ATPase (PMCA) pumps to restore [Ca^2+^]_i_ to basal levels [38].

Ca^2^+ signals in epithelial cells are highly polarized. This polarization is caused by the expression of the polylactic secretion receptor and Ca^2+^ signal transduction protein. For instance, polarized expression of Ca^2+^-signaling proteins, such as IP3Rs [39–41], SERCA and PMCA pumps [42, 43], TRPC [44] and Orai channels, and STIM1 [45, 46], has been demonstrated in epithelial cells.

Ca^2+^ signaling induces a physiological agonist concentration during Ca^2+^ oscillations that periodically results in recurrent Ca^2+^ signaling. The frequency and amplitude of the oscillations are determined by the intensity of the stimulus [47]. The direct binding of [Ca^2+^]_cyt_ to the target transporter can regulate its function, as has been demonstrated in Ca^2+^ activation of Cl^-^ channel (CaCC) [48]. Additionally, increasing [Ca^2+^]_i_ can regulate the function of target Ca^2+^ signaling proteins, such as calmodulin and Ca^2+^-calmodulin-dependent protein kinase (CamKs). Finally, Ca^2+^ evokes receptor-mediated Ca^2+^ signaling agonists that can regulate membrane transporters through the production of by-products, such as diacylglycerol (DAG) and IP3. Protein kinase C (PKC) mediates DAG activation or IP3-induced release of the IP3-binding protein with IP3 (IRBIT) from IP3Rs, which has been shown to regulate the number of epithelial transporters [49, 50].

### Ca^2+^ regulatory mechanisms of intestinal HCO_3_^−^ secretion

**Figure 1:**
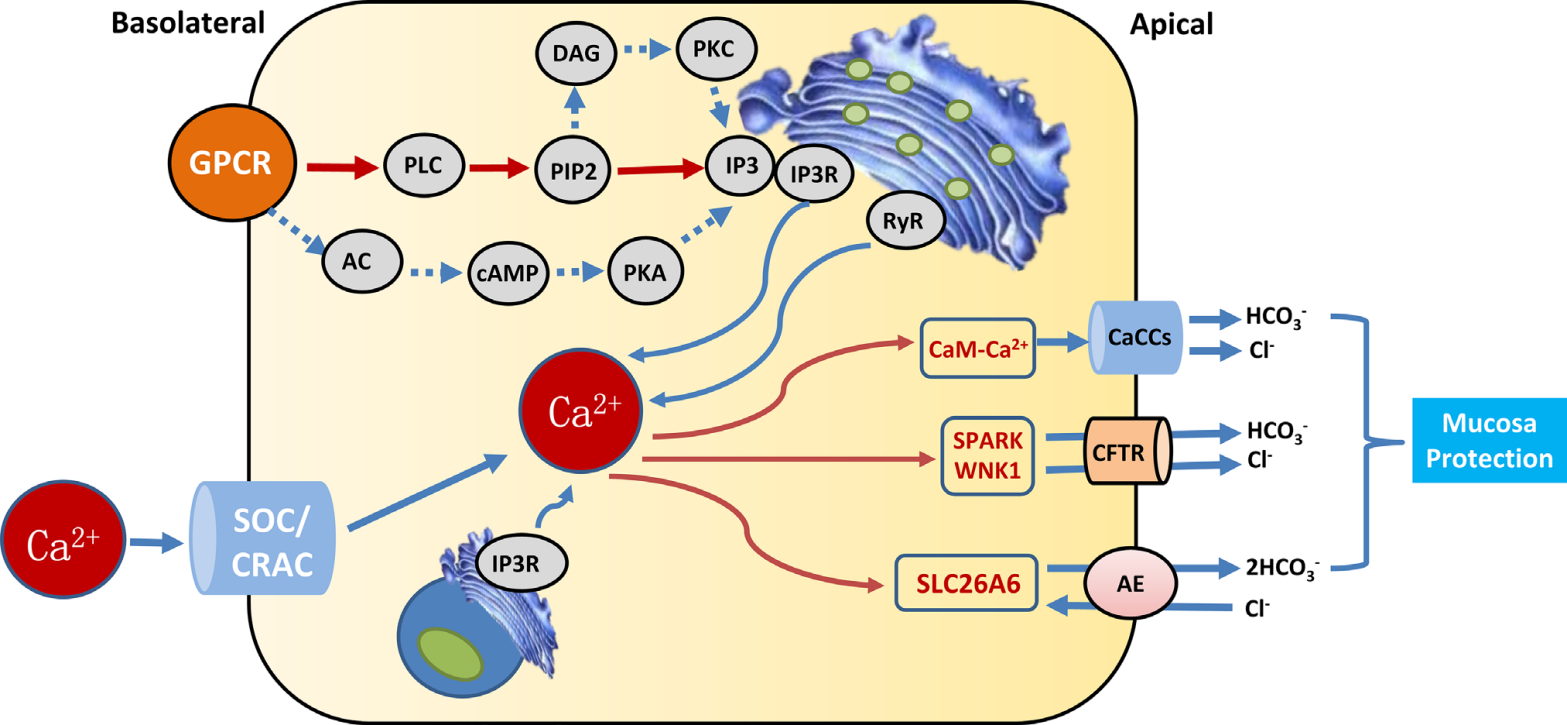
Calcium signaling that augment epithelial HCO_3_^−^ secretion G protein-coupled receptors (GPCRs), phospholipase C (PLC), phosphatidylinositol (4,5) bisphosphate(PIP2), inositol 1,4,5-trisphosphate (IP3), IP3 receptors (IP3Rs), adenylate cyclase(AC), cyclic adenosine monophosphate(cAMP), protein kinase A(PKA), diacylglycerol (DAG), protein kinase C(PKC), ryanodine receptor(RyR), Ca^2+^ release activated Ca^2+^ channel (CRAC), store-operated channels (SOCs), Ca^2+^-activated Cl^−^ channel (CaCC), cystic fibrosis transmembrane conductance regulator (CFTR), with-nolysine kinase 1 (WNK1), STE20/SPS1-related proline/alanine-rich kinase (SPAK).

Many studies have shown that epithelial HCO_3_^–^secretion plays an important role at all levels of the gastrointestinal tract from the esophagus to the colon (especially the pancreas) and that abnormal HCO_3_^–^ secretion is associated with many diseases in these organs. Of the intestinal segments, laryngeal HCO_3_^–^ secretion has been most extensively studied and defined in the duodenum. Active HCO_3_^-^ secretion is the key to protecting the epithelial cell surface against the toxic and acidic gastric contents. (i) 5-Hydroxytryptamine and ATP: The main stimulus that causes duodenal mucosal HCO_3_^–^ secretion is luminal acid, and exposure to luminal acid activates neurons to reflexively induce duodenal HCO_3_^–^ secretion [11, 12, 51, 52]. (ii) Gas: The effects of the gas medium (NO, H_2_S, and CO) on the regulation of duodenal HCO_3_^–^ secretion have been studied. Acidified mucosal release may occur systemically instead of locally, possibly through activation of capsaicin-sensitive neurons. Additionally, a large number of neurotrophic factors are not involved in laryngeal acid-induced HCO_3_^–^ secretion. These factors include capsaicin-sensitive neurons, peptides, PGs, NO, CO, and H_2_S gaseous media. Notably, many of these regulators also induce intracellular Ca^2+^ signaling in duodenal mucosal epithelial cells either directly or indirectly. For example, NO and CO can lead to the production of prostaglandin E2 (PGE2) through cyclooxygenase and cGMP-mediated activation, which in turn suggests that Ca^2+^ signaling activates Gq-coupled prostaglandin EP3 receptors [53]. (iii) Hormones: The circulating hormone CCK is released in response to the duodenal and stomach contents through postprandial duodenal HCO_3_^–^ secretion via strong induction of Ca^2+^ signaling [54]. Acid-sensitive ion channel functions represent duodenal epithelial cells and can be stimulated by acid-stimulated HCO_3_^–^ through the Ca^2+^ signaling pathway [55]. (iv) CFTR: CFTR expression is essential for HCO_3_^–^ secretion by most of the gastrointestinal epithelial tissue; in these epithelial cells, a large proportion of the transport of transgene material, including HCO_3_^–^, is mediated through the electrical diffusion pathway, suggesting that anion channels are involved in this process. CFTR anion channel activity and CFTR-dependent Cl^-^/HCO_3_^–^ exchangers appear to play important roles in all forms of duodenal HCO_3_^–^ secretion. CFTR mainly activates Ca^2+^ receptor agonists that can partially activate or enhance cAMP-mediated activation of CFTR through PKC-mediated phosphorylation or release from IP3R IRBIT through the signaling pathway. In fact, cytoplasmic Ca^2+^ signaling induces the activation of cholinergic receptors, purine receptors, Toll-like receptors, and gaseous media, which can induce CFTR activation in the duodenum through PGE2 production [56]. PGE activates the EP4 receptor in the duodenum, which eventually evokes cAMP signals and activates CFTR. Ca^2+^-induced activation of large conductance and intermediate conductance K+ channels [57] and basolateral nuclear biochemical activity [58] and the Ca^2+^-induced inhibition of apical NHE activity [59, 60] have been shown to contribute to HCO_3_^–^ secretion in parts of the intestine. (v) Calcium-sensing receptor (CaSR): The CaSRs participate in the regulation of intestinal secretion and absorption of Ca^2+^, organic nutrients, and amino acids [61, 62]. The intestinal brush border expresses CaSRs, which help detect the presence of calcium in the cavity and modify the cross-side cells to absorb calcium in cooperation with the vitamin D system [63]. The presence of CaSRs provides a basic mechanism for the detection of Ca^2+^ by intestinal cells and their responses to Ca^2+^-related biological behavior, such as intestinal secretion and uptake. In addition to the lumen in the acid-induced mechanism, the lumen of the bacterial component can induce HCO_3_^–^ secretion by the duodenal mucosa to arouse cytoplasmic Ca^2+^ signaling. (Figure 1)

### Ca^2+^ regulation of pancreatic HCO_3_^−^ secretion

In the pancreas, acinar cells secrete initial fluids that are rich in digestive enzymes and Cl^-^. In humans and several other species, HCO_3_^–^ concentrations in the pancreatic juice reach very high levels during stimulated secretion [64, 65]. Previous studies have shown that digestive enzymes secreted by receptor agonists and Ca^2+^ signaling play major roles in acinar cells, and those associated with cAMP signaling play a major role in HCO_3_^–^ secretion from duct cells [38]. Apical Ca^2+^ signals are physiologically important as they activate Ca^2+^-sensitive Cl^-^ channels, which are exclusively present in the apical membrane and are crucial for acinar fluid secretion. In pancreatic tissues, these functions act predominantly on muscarinic M3 receptors and the circulating hormone CCK, which act on CCK1 receptors. CCK also evokes Ca^2+^ spikes, albeit with a somewhat different pattern from that generated by ACh. Although all Ca^2+^ spikes can be blocked by IP3R or RyR antagonists irrespective of whether they are evoked by ACh or CCK, the action of ACh appears to be initiated by phospholipase C activation via IP3 generation. The higher pancreatic juice HCO_3_^–^ concentration is transported by CFTR, which acts as a HCO_3_^–^ channel. The activities of these transporters are directly or indirectly affected by Ca^2+^ signaling; thus, cytoplasmic Ca^2+^ and PKC can activate the CFTR-dependent Cl^-^/HCO_3_^–^ exchange [66] and CFTR anion channel activity [50].

### Epithelial restitution of GI protection

### Role of epithelial restitution in the intestine

The small intestine is the main organ involved in the absorption and secretion nutrients by adjusting the flow of water and several ions (Na+, Cl^-^, K+, and Ca^2+^) to maintain the water and electrolyte balance. Therefore, the integrity of the intestinal mucosa is very important. Epithelial return is a highly regulated process that relies on energy and is involved in intracellular and extracellular signals and tissue repair via biomolecules. This repair process involves PGs, cytoskeletal rearrangement, ion transporters, and other cellular processes [67–70]. In response to acute interruption of gastric epithelial cells, cell migration is the first response towards the restoration of epithelial continuity and barrier function [71]. Recently, all gastrointestinal epithelial cells have been shown to secrete HCO_3_^–^, which plays an important role in protection against epithelial cell damage from the luminal contents, such as gastric acid, drugs, reactive oxygen species, bile acids, and bacterial products in the esophagus, stomach, and small intestine [1, 2, 72], trypsin, bile acids, and alcohols in the pancreatic ducts [4], and toxic bile acids in the biliary tract [73]. In the lower digestive tract, bicarbonate secretion not only protects the intestinal mucosal barrier but also is the most important mechanism regulating the acid/alkaline balance apart from kidney function. CFTR is obviously expressed in the gastrointestinal epithelium in most tissues, including the esophagus [6], small intestine [27], biliary tract [74, 75], and pancreatic duct [38], as well as the reproductive tract [76–79], which creates the necessary conditions for the airline HCO_3_^–^ secretion.

### Ca^2+^ signaling in epithelial restitution

Ca^2+^ is the second messenger of numerous cellular processes, including the effects of gastric acid/bicarbonate secretion, mucus secretion, and cell migration. We have shown that cytoplasmic Ca^2+^ mobilization in gastric epithelial cells occurs during the return of the small molecule signal to repair the central signal. However, extracellular Ca^2+^ is also mobilized in the upper part of wounded juxtamucosal lumen spaces [80], and evidence suggests that extracellular Ca^2+^ is the third messenger and thus also promotes restoration of the gastric epithelium. Intracellular and extracellular Ca^2+^ interactions are necessary for efficient gastric epithelial restitution. The human intestinal mucosal epithelia contains a Ca^2+^ sensing mechanism. In the early 1980s, changes in extracellular Ca^2+^ and modulation of the 1, 25-dihydroxyvitamin D3 levels were observed during regulated uptake and/or Ca^2+^ secretion in isolated rat colonic mucosal cells. Subsequently, there was a 30-year interval with virtually no *in vivo* studies exploring the role of [Ca^2+^]_i_ in gastric epithelial cells.

The most important ion channels are Cl^-^/HCO_3_^–^ exchangers, and in many epithelial tissues, including the pancreatic ducts, salivary gland ducts, and the duodenum, apical HCO_3_^−^ secretion is frequently associated with Cl^−^ absorption [81]. In humans and other mammals, which encode the SLC4 and SLC26 family gene products involved in Cl^-^/HCO_3_^–^ exchange activity, recent evidence suggests that drug transporter SLC26 family members can mediate Cl^-^/HCO_3_^–^ exchange. CaCCs can also mediate electro diffusive HCO_3_^−^ transport in the apical epithelial membrane. Recently, members of the anoctamin family (ANO; also known as TMEM16), especially ANO1/TMEM16A and ANO2/TMEM16B, have been shown to function as CaCCs in the intestine, trachea, salivary glands, and olfactory organ [82–86]. Ca^2+^-induced activation of CaCCs has been suggested to contribute to HCO_3_^−^ secretion in some epithelial tissues. CFTR is a cAMP-activated anion channel that is mutated in CF [87]. CFTR expression is a necessary condition for HCO_3_^–^ secretion by most GI and airway epithelial cells [81]. Among these epithelial cells, a large part of the transgene material from HCO_3_^–^ transportaccumulates through the electro diffusive pathway, suggesting that the anion channel is involved in this process.

### The underlying mechanisms

Despite the exciting potential shown by the results discussed above, few reports have measured Ca^2+^ in the gastric epithelia. Intracellular loading of conventional acetoxymethyl ester Ca^2+^-sensitive fluorescent probes has been used to study this topic. In 1997, the gene encoding yellow cameleon (YC) protein was discovered; subsequently, cyan fluorescent protein (CFP) was developed, and yellow fluorescent protein (YFP) was associated with the M13 calmodulin-binding domain and calmodulin. YC transgenic mice have been created, which allows direct observation of [Ca^2+^] in real time [88].

Eitaro Aihara and Marshall H Montrose's work and the work of others has shown that there is a pH microdomain adjacent to the surface of the epithelium that is altered in the presence of epithelial damage [80–89]. Based on these advances in our knowledge, the conceptual and experimental foundation for evaluating luminal Ca^2+^ microdomains has been solidified in recent years. These studies used two-photon confocal microscopy to investigate the gastric epithelial restitution model.

In the case of gastric mucosal protection, bicarbonate secretion is mediated by the EP1 receptor via a mechanism mediated by verapamil [13]. These data suggest that an increase in epithelium recovery in [Ca^2+^]_i_ may mediate PGE2 activation via PLC/IP3 upstream of the EP1 receptor. Additionally, *in vitro* studies of gastric epithelial cells have reported that PGE2 is released by PLC inhibitors, suggesting that an increase in [Ca^2+^]_i_ in response to damage enhances PGE2 production via the late maintenance cycle, which is expected to stimulate repair while maintaining high Ca^2+^ levels [90, 91]. Evidence from the use of inhibitors suggests that some of the Ca^2+^ influx important for cell migration occurs through voltage-gated Ca^2+^ channels *in vivo* [92]. Other Ca^2+^ channels, such as transient receptor potential (TRP) channels, may also regulate the Ca^2+^ influx. TRPC appears to serve as a store for the Ca^2+^ channels (SOC) in many cells, but the transnational radical subtype expression profiles of gastric epithelial cells are still unknown [93, 94]. Recently, TRPC has been shown to associate with Orai1 and STIM1 in several models [95–97]. However, due to lack of study of gastric epithelial cells or other areas of the gastrointestinal tract, the mechanism underlying the Ca^2+^ influx in gastric epithelial cells is unknown.

The key early observation was that the chelating activity of extracellular Ca^2+^ reduced the potential difference of the gastric mucosa. Recent reports have shown that Ca^2+^ release into the gastric gland can occur as part of the normal physiological functions of regulation. The extracellular Ca^2+^ gradient appears to be present in the various medial gastric lumen compartments, and this Ca^2+^source may at least have physiological effects that promote mucus and HCO_3_^–^ secretion. Secretion from intact tissue is one component of the first line of gastric defense. Extracellular Ca^2+^ also plays a role in injured tissue. Increased luminal Ca^2+^ benefits epithelial repair and is dependent on [Ca^2+^]_i_ increases, which most likely results from the active Ca^2+^ efflux from surviving epithelial cells as a result of epithelial cell repair.PMCA1 has been reported to be essential for the routine maintenance of intracellular Ca^2+^ homeostasis, whereas PMCA4 performs specialized physiological functions [98]. PMCA1 is reported to have an important effect on gastric restitution and the regulation of extracellular Ca^2+^ following injury [99]. Since the lateral cell membrane is exposed to light lesions in the gastric cavity and interruptions of epithelial continuity, enhanced permeability is the easiest way to predict the microenvironment that will allow observation of high Ca^2+^ concentrations at the site of injury.

## CONCLUSIONS

In conventional signaling models, most physiological changes are triggered by intracellular second messengers, such as cAMP, cGMP, and Ca^2+^. Ca^2+^ is the most important signaling molecule involved in epithelial restitution; beyond HCO_3_^–^ secretion, the promotion of intestinal epithelial restitution becomes the primary barrier against epithelial damage. We can apply these new perspectives to drug development; however, whether the activation of receptors that have recently been implicated in stimulating epithelial HCO_3_^–^ secretion may be feasible and whether this approach will provide therapeutic benefits are unknown. The development of small molecules targeting CFTR, regulatory proteins, or stimulatory receptors suggest that such strategies may become available in the future.
